# Heart failure mortality is inversely related to wealth of country

**Published:** 2017

Death rates in patients with heart failure is inversely related to the wealth of the country they live in, according to late breaking results from the INTERCHF study, presented recently at Heart Failure 2017 and the 4th World Congress on Acute Heart Failure. Death rates in India and Africa were three to four times higher than those documented in Western countries.

‘Heart failure is a common condition that causes morbidity and mortality worldwide’, said lead author Dr HishamDokainish, a principal investigator at the Population Health Research Institute (PHRI), McMaster University, Hamilton, Canada.

‘Most data on heart failure have come from Western countries but the majority of the world’s population lives elsewhere’, he continued. ‘This study was conducted to fill large gaps in knowledge about congestive heart failure in non-Western countries.’

The International Congestive Heart Failure (INTERCHF) study was an observational cohort study that enrolled 5 823 patients with heart failure in 16 countries grouped into six regions: Africa (Mozambique, Nigeria, South Africa, Sudan, Uganda), China, India, the Middle East (Egypt, Qatar, Saudi Arabia), south-east Asia (Malaysia, the Philippines), and South America (Argentina, Chile, Colombia and Ecuador).

Data on each patient was collected at baseline, six months and one year, and entered into the electronic data-management system at PHRI. Baseline data included demographics (age, gender), cardiac and non-cardiac factors (previous heart attack or stroke, duration of congestive heart failure, diabetes mellitus, renal failure, chronic obstructive pulmonary disease), medications, socio-economic factors (education level, literacy, employment, urban/rural setting) and heart failure aetiology.

At six months and one year, data were collected on the frequency and cause of any hospitalisations in the previous six months. Information was also recorded on death and cause of death. The investigators calculated death rates in each region and adjusted for 20 variables, which included demographic, clinical, and socio-economic factors, medications and cause of heart failure.

The overall all-cause mortality rate for the entire study population was 17%. It was highest in Africa (34%) and India (23%), intermediate in south-east Asia (15%), and lowest in the Middle East (9%), South America (9%) and China (7%).

Dr Dokainish said: ‘Mortality in patients with heart failure was inversely related to the wealth of the country. The poorer the country, the higher the mortality rate, and the richer the country, the lower the mortality rate.’

‘In Western countries the one-year mortality rate for patients with heart failure is 5–10%’, added Dr Dokainish. ‘We’re finding two to three times that death rate in African and Indian patients.’

‘We were very surprised by the much higher mortality rates’, he continued. ‘You could say maybe the patients in Africa or India were sicker, or didn’t take their medicines, or had poorer heart function, but we adjusted for all of those things and don’t really understand why their death rates were so much higher.’

The researchers hypothesised that variables not measured in the study contributed to the high death rates, such as access to and quality of healthcare, and cardiac biomarkers. These variables will be measured in the next phase of the research programme, the Global Congestive Heart Failure (G-CHF) study, which aims to recruit 25 000 heart failure patients from all inhabited continents and income levels. Genetic analyses will also be conducted in a G-CHF sub-study.

Dr Dokainish said: ‘INTERCHF has shown that there are large differences in the risk of heart failure patients dying at one year depending on where they live. We hope to discover why these differences exist through the G-CHF study. If that identifies barriers to receiving care that are due to the way a healthcare system is structured, access to healthcare, or quality of healthcare, then that would need to be addressed.’

## References

[1] European Society of Cardiology Press Office.

